# Lymphocytes upregulate CD36 in adipose tissue and liver

**DOI:** 10.1080/21623945.2019.1609202

**Published:** 2019-04-30

**Authors:** Jacob Couturier, Alli M. Nuotio-Antar, Neeti Agarwal, Gregory K. Wilkerson, Pradip Saha, Viraj Kulkarni, Samir K. Lakhashe, Juan Esquivel, Pramod N. Nehete, Ruth M. Ruprecht, K. Jagannadha Sastry, Jennifer M. Meyer, Lori R. Hill, Jordan E. Lake, Ashok Balasubramanyam, Dorothy E. Lewis

**Affiliations:** a Division of Infectious Diseases, Department of Internal Medicine, The University of Texas Health Science Center at Houston, Houston, TX, USA; b US Department of Agriculture/Agricultural Research Center, Children’s Nutrition Research Center, Department of Pediatrics, Baylor College of Medicine, Houston, TX, USA; c Division of Diabetes, Endocrinology and Metabolism, Department of Medicine, Baylor College of Medicine, Houston, TX, USA; d Department of Comparative Medicine, The University of Texas MD Anderson Cancer Center, Bastrop, TX, USA; e Department of Virology and Immunology, Texas Biomedical Research Institute, San Antonio, TX, USA; f Southwest National Primate Research Center, Texas Biomedical Research Institute, San Antonio, TX, USA; g Department of Microbiology, Immunology & Molecular Genetics, The University of Texas Health Science Center at San Antonio, San Antonio, TX, USA; h Department of Immunology, The University of Texas MD Anderson Cancer Center, Houston, TX, USA; i Department of Veterinary Medicine and Surgery, The University of Texas MD Anderson Cancer Center, Houston, TX, USA

**Keywords:** Adipose tissue, CD36, metabolism, obesity, T cells

## Abstract

CD36 is a multifunctional scavenger receptor and lipid transporter implicated in metabolic and inflammatory pathologies, as well as cancer progression. CD36 is known to be expressed by adipocytes and monocytes/macrophages, but its expression by T cells is not clearly established. We found that CD4 and CD8 T cells in adipose tissue and liver of humans, monkeys, and mice upregulated CD36 expression (ranging from ~5–40% CD36+), whereas little to no CD36 was expressed by T cells in blood, spleen, and lymph nodes. CD36 was expressed predominantly by resting CD38-, HLA.DR-, and PD-1- adipose tissue T cells in monkeys, and increased during high-fat feeding in mice. Adipose tissue and liver promote a distinct phenotype in resident T cells characterized by CD36 upregulation.

## Introduction

Adipose tissue is a highly metabolic and lipid-rich microenvironment that may regulate resident immune cell function and homeostasis differently compared to other tissues such as blood and lymphoid tissues, resulting in distinctive phenotypes and functions. The infiltration of immune cells, particularly macrophages and T cells, into adipose tissue and regulation of adipocyte functions have been extensively characterized. Less is known with respect to markers of immune cells specific for adipose tissue migration and residence – such information would enhance understanding of T cell phenotypes and function in adipose tissue.

CD36 is a multifunctional receptor for lipid uptake and a scavenger receptor that is widely expressed by adipocytes and antigen-presenting cells including dendritic cells, monocytes and macrophages [1–4]. CD36 expression by T cells is less well characterized. Zamora et al. elegantly showed that detection of CD36 on human blood T cells is spurious, due mainly to interaction with contaminating platelets which express CD36 robustly [5]. In adipose tissue of obese mice, CD36 is expressed by CD4+ Tregs and promotes Treg survival [6,7]. Han et al. conducted RNA-seq studies of memory CD8 T cells isolated from different tissues in mouse models of bacterial infections and observed upregulation of lipid biosynthesis/efflux pathways with increased CD36 and FABP4 expression in CD8 T cells from mesenteric adipose tissue compared to spleen and intestinal CD8 T cells [8]. Additionally, Pan et al. demonstrated upregulation of CD36 and FABP4/FABP5 mRNA expression in skin tissue-resident memory CD8+ T cells (Trm) during viral infections, which enhanced Trm function and survival [9]. These data suggest the hypothesis that CD36 signalling may be an important regulator of T cell function and homeostasis in adipose tissue.

Expression of CD36 and fatty acid uptake are linked to a number of diseases and pathologies. For example, increased expression of CD36 in adipocytes and adipose tissue macrophages is associated with obesity and inflammation [10–14]. CD36 can be upregulated in cancer cells by adipocytes and high-fat diet to increase fatty acid uptake and promote tumour growth and metastases [15–17]. Here we show that CD36 is upregulated by T cells uniquely in adipose tissue and liver, and demonstrate that platelet discrimination is important when determining CD36 expression by T cells in highly vascularized tissues.

## Materials and methods

### Human subjects, non-human primates and ethics

For studies of human cells, tissue samples were obtained from deceased (within 24 h of death) de-identified donors through the National Disease Research Interchange (NDRI – Philadelphia, PA). For studies of monkey cells, tissues were obtained from necropsied rhesus macaques (within 24 h of necropsy) through the Tissue Share Program at the Southwest National Primate Research Center (San Antonio, TX) or at the MD Anderson Cancer Center Keeling Center for Comparative Medicine and Research (Bastrop, TX). The use and collection of samples from rhesus macaques were reviewed and approved by the Texas Biomedical Research Institute IACUC and the MD Anderson Cancer Center IACUC.

### Tissue processing and single cell preparations

Human and monkey tissues were processed into single cell preparations for flow cytometry measurements. PBMC was isolated from anti-coagulated blood by density-gradient centrifugation with Ficoll-Paque PLUS (GE Healthcare). Adipose tissue samples were minced, then digested with 1mg/ml collagenase II (Worthington) for 1–2 h at 37°C. Cells were then centrifuged to separate stromal-vascular-fraction (AT-SVF) cells from adipocytes in the floater fraction. AT-SVF cells were washed with PBS/2%FBS and filtered through 100 µm strainers. Lymph nodes were minced and incubated with 1mg/ml collagenase II and 0.1mg/ml DNase for 1–2 h at 37°C. Cells were washed with PBS/2%FBS and filtered through 100 µm strainers. Pieces of spleen or liver were minced and cells washed with PBS/2%FBS. Red blood cells were lysed with NH_4_Cl lysis buffer, then cells washed with PBS/2%FBS and filtered through strainers.

### Flow cytometry

For flow cytometry measurements of single cells prepared from human and monkey tissues, 1 × 10^6^ cells were washed with PBS/2%FBS, incubated with 1–3 μg/ml antibodies for 60 min at 4°C and washed with PBS/2%FBS. Cells were analysed with a Gallios Flow Cytometer and Kaluza software (Beckman-Coulter). The following monoclonal antibodies specific for human and rhesus macaques were from Biolegend or BD Biosciences: CD3-(FITC, PE, PerCPCy5.5, or APCCy7), CD4-(PE, APC, PECy5, or AF700), CD8-(FITC, PE, or PerCPCy5.5), CD25-PE, CD38-AF647, HLA.DR-(FITC, PE, or APC), PD-1-PECy7, and CD41-APCCy7. Anti-human CD36-FITC mabs (clone FA6-152) was from Stemcell Technologies.

For the C16-BODIPY uptake assay (fluorescent palmitate analogue from Invitrogen) with human AT-SVF cells, 1 × 10^6^ cells were first stained with surface antibodies for 30 min. Cells were washed and pre-incubated with either 50μl Palmitate-BSA solution (Agilent Technologies), 0.5μM sulfosuccinimidyl oleate – SSO (Cayman Chemical), or no treatment, for 15 min in 1 ml RPMI medium at 37°C. 0.1 μM C16-BODIPY was then added for an additional 10 min. Cells were washed with ice-cold PBS to stop the reaction and analysed with flow cytometer. Negative/positive signals were determined by appropriate isotype or fluorescence-minus-one (FMO) controls.

### Murine studies

For high-fat diet mouse studies, C57BL/6J breeding pairs were obtained from The Jackson Laboratory. Littermate males were housed and bred in a pathogen-free facility with constant temperature of 20–22°C, 12/12 hr light/dark cycle, and free access to food and water. At 6 weeks of age, mice were divided into two weight-matched dietary treatment groups and either continued on standard chow diet (Harlan-Teklad 2920X) or fed a 21% milk fat, 34% sucrose Breslow Western-type diet (TestDiet 5TFH) for 14 weeks, then mice sacrificed. Body weights were measured once per week, and whole-body fat mass and lean mass were assessed with an EchoMRI Whole Body Composition Analyser (Echo Medical Systems) during the last week of the diet study. Prior to tissue harvest, mice were euthanized between 6:00 am and 12:00 pm via isoflurane inhalation followed by cervical dislocation and perfusion with 20 mL PBS to remove circulating blood cells from tissues. The mouse study was conducted following approval of protocols by the Baylor College of Medicine IACUC.

Tissues were harvested and processed into single cell preparations as described above for flow cytometry measurements. Cells were washed with PBS/2%FBS, then incubated with antibodies for 30 min at 4°C. Cells were washed and analysed with flow cytometer. CD3-(FITC or APC), CD4-PerCPCy5.5, CD36-PE, CD41-Pacific blue, and PD-1-APCCy7 antibodies, and Zombie yellow viability dye were from Biolegend. For measurement of C16-BODIPY palmitate uptake by AT-SVF cells, 5 × 10^5^ cells were first stained for surface markers for 30 min, washed, then incubated with 0.1μM C16-BODIPY for 10 min in 1 ml RPMI medium at 37°C. Cells were washed with ice-cold PBS and analysed with flow cytometer.

### Statistics

Data were analysed with Excel and GraphPad Prism. Comparisons between conditions were conducted with Student’s t-test and p < 0.05 was considered significant.

## Results

### T cells upregulate CD36 in human adipose tissue

**Figure 1.**
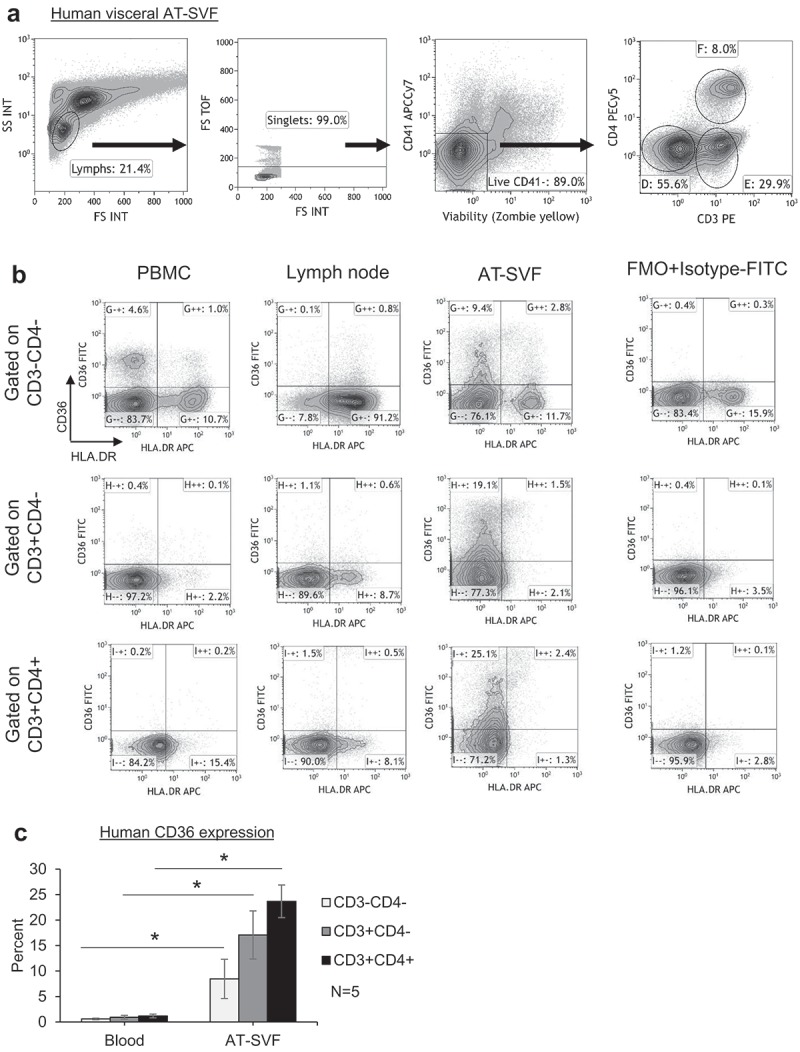
**CD36 expression by human adipose tissue lymphocytes**. Single cells were prepared from blood, inguinal lymph node, and visceral adipose tissue stromal-vascular-fraction (AT-SVF) cells of a human donor. (a) Shown is a sample flow cytometry gating scheme of AT-SVF lymphocytes (CD3-CD4-, CD3+CD4-, and CD3+CD4+ cells, with CD41 used as a platelet discriminator in the third top dotplot from the left), and (b) HLA.DR/CD36 dotplots gated on CD3-CD4-, CD3+CD4-, and CD3+CD4+ lymphocytes of PBMC, lymph node, and AT-SVF cells, with CD36 expression based on the FMO+Isotype-FITC control. (c) Mean±sem CD36 expression by CD3-CD4-, CD3+CD4-, and CD3+CD4+ lymphocytes of human PBMC and AT-SVF cells (*p < 0.05, N = 5).

CD36 expression was first studied in human adipose tissue T cells by flow cytometry. Figure 1(a,b) shows the flow cytometry gating scheme for examination of CD36 expression in conjunction with HLA.DR (a T cell activation marker) by blood, lymph node, and visceral AT-SVF T cells of a human donor. As CD36 is highly expressed by platelets that can bind to blood T cells during PBMC isolation, CD41 (a platelet marker that is not expressed by T cells) was included as a platelet discriminator [5]. AT-SVF T cells clearly showed the highest expression of CD36, in which at least 20% of AT-SVF CD3+CD4- and CD3+CD4+ T cells were CD36+ (which was predominantly expressed by resting HLA.DR- cells), compared to <1% CD36 expression by PBMC and lymph node T cells. Examination of blood and subcutaneous AT-SVF T cells from five human donors in Figure 1(c) showed significantly increased CD36 expression by AT-SVF T cells, ~17–23% CD36+ AT-SVF T cells compared to ~1% CD36+ PBMC T cells (p < 0.05). It was difficult to further study CD36 function in blood T cells as CD36 expression was virtually undetectable, and not inducible upon culture with a variety of treatments including CD3/CD28 costimulation, PHA, PMA/IO, stimulatory cytokines (IL2, IL7, or IL15), PPARγ agonists (rosiglitazone or pioglitazone), or co-culture with human primary adipocytes (data not shown).

To determine if adipose T cell CD36 mediates fatty acid uptake, AT-SVF cells of two human donors were cultured with the fluorescent palmitate analogue C16-BODIPY for 10 min with or without pretreatment with a CD36 chemical inhibitor SSO (Supplementary Figure 1). Cells were also pretreated with palmitate-BSA as a positive control to block C16-BODIPY uptake. Supplementary Figure 1 shows the flow cytometry gating scheme for examination of C16-BODIPY uptake by CD3-CD4-, CD3+CD4-, or CD3+CD4+ AT-SVF cells. However, SSO did not inhibit C16-BODIPY uptake in AT-SVF lymphocytes, whereas palmitate-BSA did reduce C16-BODIPY uptake, suggesting CD36 may not significantly regulate uptake of some long-chain fatty acids by adipose tissue T cells. We next further studied the CD36+ phenotype of lymphocytes in multiple tissues of monkeys and mice.

### CD36 is upregulated in adipose tissue T cells of uninfected and SIV/SHIV-infected rhesus monkeys

**Figure 2.**
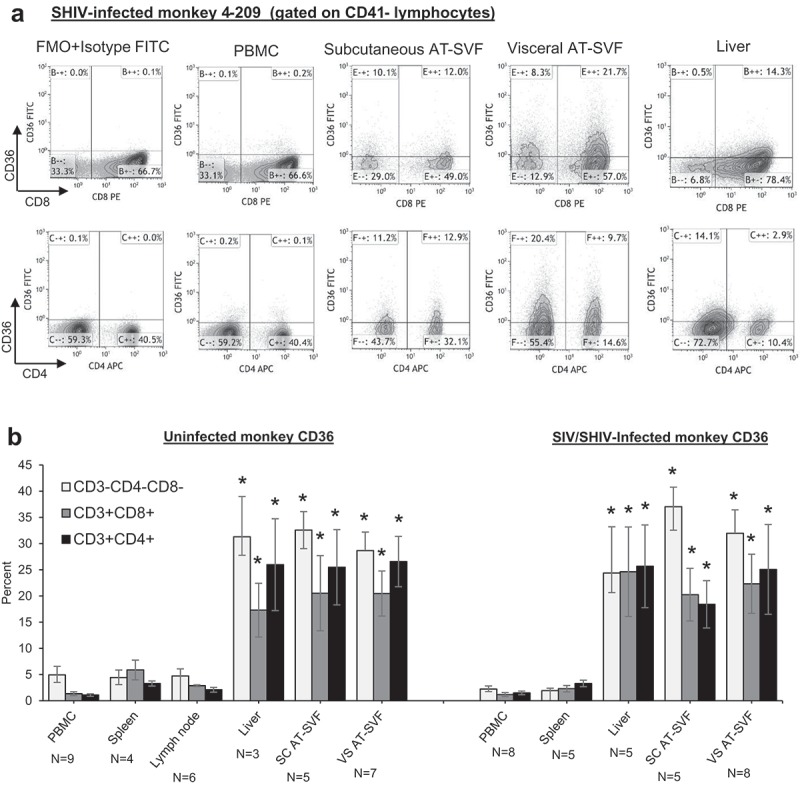
**Upregulation of CD36 expression by rhesus monkey liver and adipose tissue SVF cells**. Single cells were prepared from PBMC, spleen, lymph nodes, liver, and subcutaneous and visceral AT-SVF cells of uninfected and SIV- or SHIV-infected monkeys. CD36 expression was then examined on CD3-CD8-CD4-, CD3+CD8+, and CD3+CD4+ lymphocytes by flow cytometry. Shown in A are sample CD8/CD36 and CD4/CD36 flow cytometry dotplots (gated on live CD41- lymphocytes) of an SHIV-infected monkey, and B shows mean±sem CD36 expression (*p < 0.05 compared to PBMC, spleen, or lymph nodes; lymph nodes were not examined for infected monkeys).

**Figure 3.**
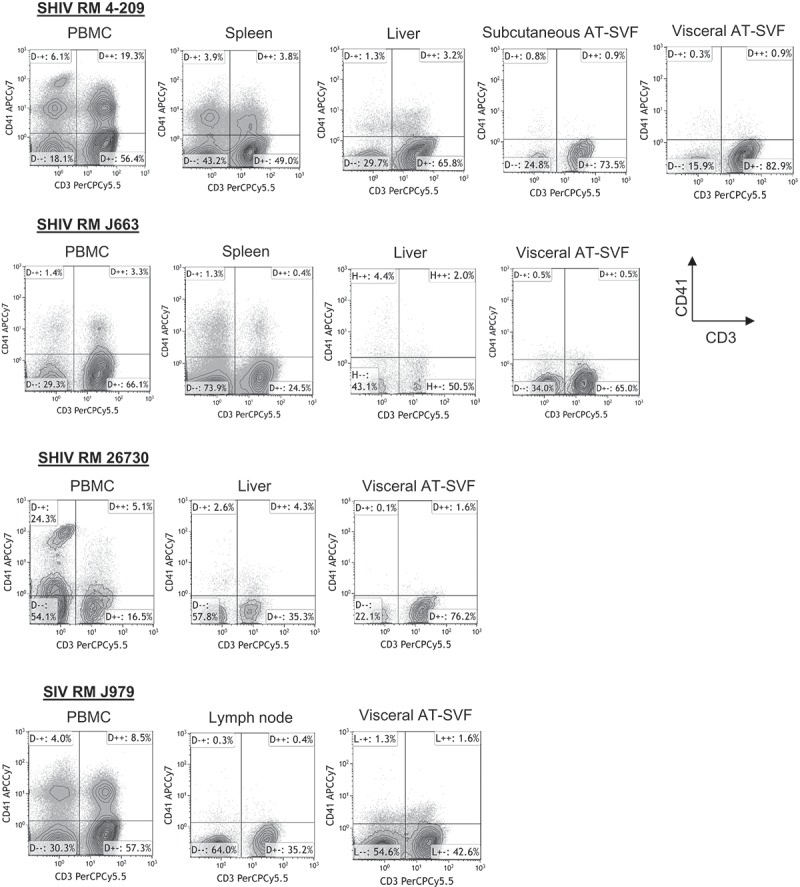
**Assessment of potential platelet interactions with T cells prepared from different tissues of monkeys**. Single cells were prepared from PBMC, spleen, lymph node, liver, or subcutaneous and visceral AT-SVF cells of infected monkeys. Cells were then examined for coexpression of the CD41 platelet marker with CD3+ T cells by flow cytometry. Shown are sample CD3/CD41 dotplots of cells (gated on lymphocytes in the light scatter plot) from different tissues of four different monkeys, indicating CD3+CD41+ cells (platelet contamination of T cells) occur mostly in PBMC and spleen preparations.

We next examined CD36 expression in adipose tissue T cells of uninfected and SIVmac251- or SHIV-sf162p3-infected rhesus monkeys (Figure 2). PBMC, spleen, lymph node, liver, and subcutaneous and visceral AT-SVF cells of 3–9 uninfected and 5–8 infected monkeys were examined for CD36 expression by flow cytometry. Figure 2(a) shows sample CD8/CD36 and CD4/CD36 flow cytometry dotplots of PBMC, AT-SVF, and liver cells from a SHIV-infected monkey, and Figure 2(b) shows mean±sem CD36 expression by CD3-CD4-CD8-, CD3+CD8+, and CD3+CD4+ cells. CD36 expression was significantly higher in liver and AT-SVF T cells (~20–30% CD36+) compared to PBMC, lymph node, and spleen cells (<5% CD36+, p < 0.05). CD36 expression by AT-SVF T cells did not significantly differ between uninfected and infected monkeys, although the infected monkeys varied with respect to viral infection (SIV or SHIV) and length of infections. CD36 expression by adipose T cells also did not differ between subcutaneous and visceral depots. Additionally, Figure 3 shows more clearly that CD41+ platelet interaction with CD3+ T cells was frequently observed with monkey PBMC, spleen, and liver T cells, whereas platelet contamination was minimal in lymph node and AT-SVF cells, emphasizing the importance of platelet discrimination when examining CD36 expression in T cells in highly vascularized tissues (shown are sample CD3/CD41 dotplots of tissues from four monkeys).

**Figure 4.**
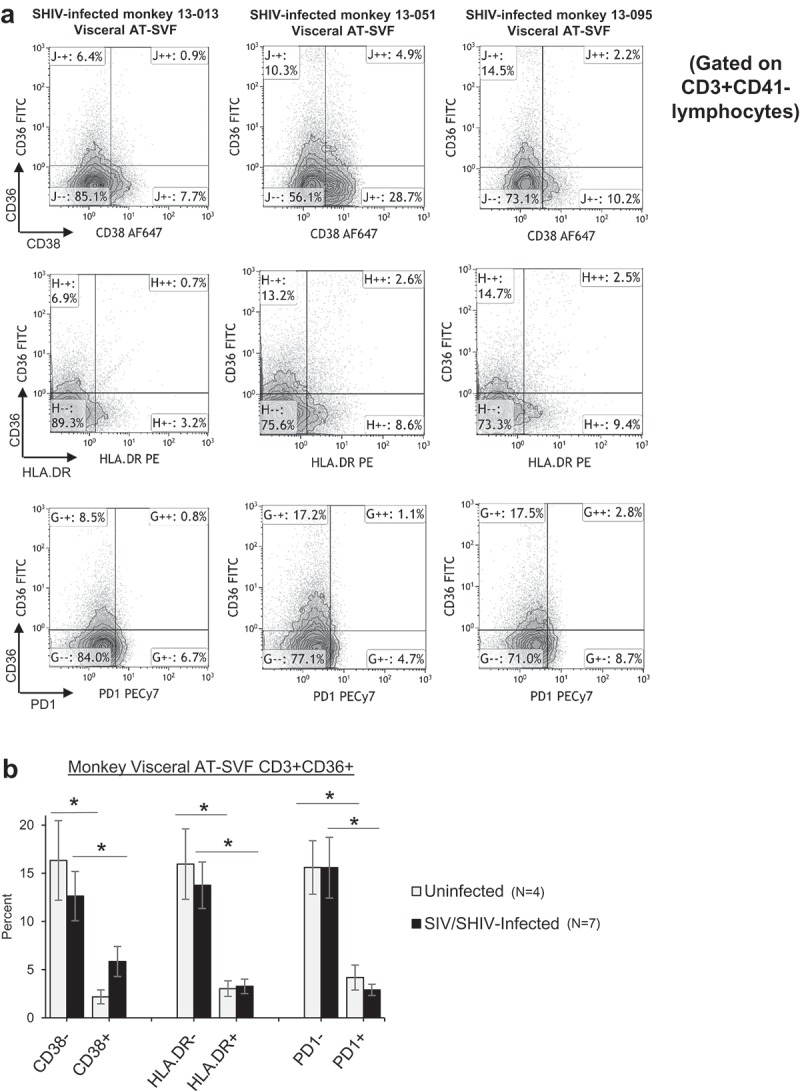
**Predominant expression of CD36 by resting T cells in adipose tissue of rhesus monkeys**. Visceral AT-SVF cells were prepared from uninfected and infected monkeys. AT-SVF CD3+ T cells were then examined for expression of CD36 in conjunction with CD38, HLA.DR, or PD-1 by flow cytometry. (a) Sample CD38/CD36, HLA.DR/CD36, and PD-1/CD36 flow cytometry dotplots (gated on CD3+CD41- T cells) of three SHIV-infected monkey AT-SVF cells. (b) Mean±sem CD36 expression by CD38-/+, HLA.DR-/+, and PD-1-/+ AT-SVF CD3+ T cells (*p < 0.05 comparing CD38-, HLA.DR-, or PD-1- cells to CD38+, HLA.DR+, or PD-1+ cells).

We next examined CD36 expression in conjunction with activation markers CD38, HLA.DR, and PD-1 in AT-SVF T cells of four uninfected and seven SIV/SHIV-infected monkeys (Figure 4). Figure 4(a) shows sample CD38/CD36, HLA.DR/CD36, and PD-1/CD36 flow cytometry dotplots of visceral AT-SVF T cells (gated on CD3+CD41- T cells) from three infected monkeys. Most CD36 was expressed by resting CD38-, HLA.DR-, and PD-1- AT-SVF T cells (~15%) compared to activated CD38+, HLA.DR+, and PD-1+ T cells in both uninfected and infected monkeys (<5%, p < 0.05, Figure 4(b)). These data suggest that CD36 is a receptor expressed distinctly by adipose tissue and liver T cells which may regulate T cell activation.

### CD36 is upregulated in adipose tissue T cells during high-fat feeding in mice

**Figure 5.**
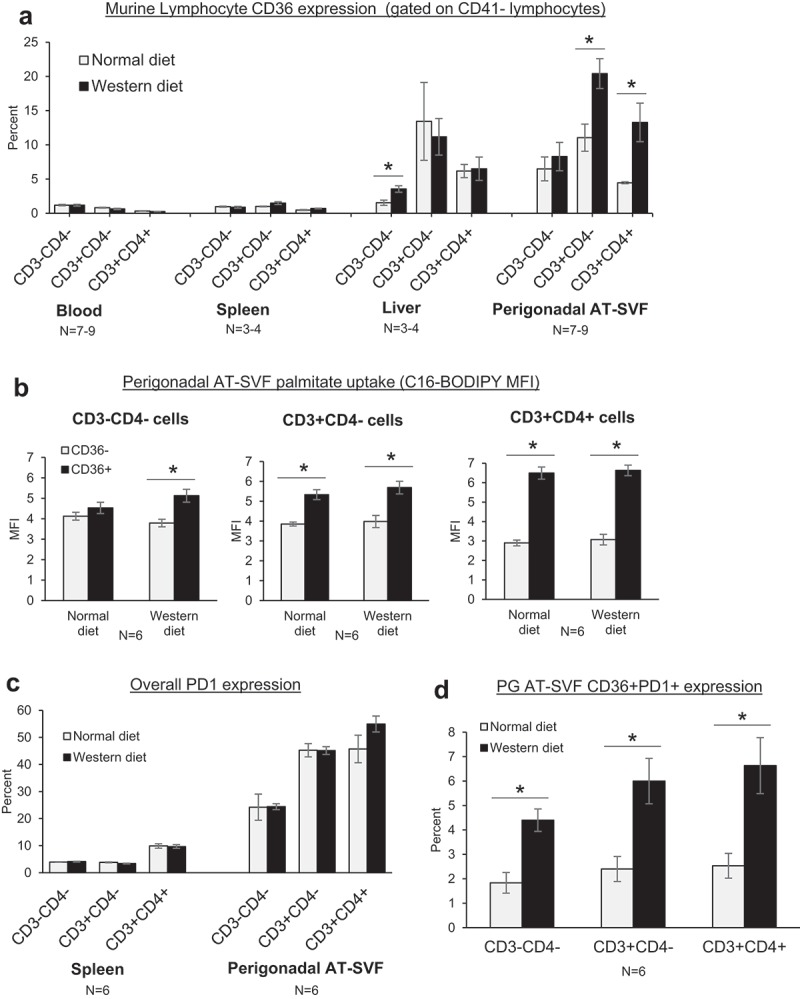
**CD36 expression in adipose tissue T cells during high-fat diet in mice**. (a) C57BL/6J mice were fed standard chow diet (Harlan-Teklad 2920X) or 21% milk fat/34% sucrose Breslow Western-type diet (TestDiet 5TFH) for fourteen weeks. Mice were sacrificed and single cells prepared from blood, spleen, liver, and perigonadal (PG) AT-SVF cells. Cells were then examined for CD36 expression on CD3-CD4-, CD3+CD4-, and CD3+CD4+ lymphocytes (gated on live CD41- lymphocytes) by flow cytometry. Shown are mean±sem CD36 expression (*p < 0.05). (b) Fatty acid uptake by adipose tissue CD36+ lymphocytes. PG AT-SVF cells were incubated with a fluorescent palmitate analog (C16-BODIPY), washed, and examined for C16-BODIPY uptake by CD36- and CD36+ cells (gated on CD3-CD4-, CD3+CD4-, or CD3+CD4+ lymphocytes). Shown are mean±sem C16-BODIPY mean fluorescence intensities (*p < 0.05). (c–d) Upregulation of CD36+PD-1+ lymphocytes in adipose tissue during western diet. Expression of CD36 and PD-1 by CD3-CD4-, CD3+CD4-, and CD3+CD4+ lymphocytes of spleen and PG AT-SVF cells were examined by flow cytometry. Shown are mean±sem overall PD-1 expression by spleen and PG AT-SVF lymphocytes (c), and mean±sem CD36+PD-1+ double-positive lymphocytes in PG AT-SVF cells (d, *p < 0.05).

We lastly examined CD36 expression in adipose tissue T cells of mice in relation to high-fat feeding (Figure 5). Six weeks-old C57BL/6J male mice were fed normal chow or high-fat western diet for 14 weeks, then tissues harvested and processed into single cells for flow cytometry measurements. As with the monkeys, CD36 expression by liver and perigonadal AT-SVF lymphocytes (CD3-CD4-, CD3+CD4-, and CD3+CD4+) was upregulated compared to blood and spleen T cells (p < 0.05, Figure 5(a)). Intriguingly, CD36 expression by AT-SVF T cells was increased further by western diet compared to normal diet (p < 0.05). However, CD36 expression by liver T cells was unaffected by western diet, despite the higher liver fat content as shown in Supplementary Figure 2, indicating that the tissue compartments are different. Additionally, examination of palmitate uptake (C16-BODIPY) by CD36- and CD36+ AT-SVF lymphocytes in vitro showed greater uptake of palmitate by CD36+ compared to CD36- cells (p < 0.05), although palmitate uptake was not affected by western diet (Figure 5(b)). Lastly, CD36 was examined in conjunction with PD-1 (Figure 5(c,d)). Compared to splenocytes, expression of PD-1 was substantially elevated by PG AT-SVF lymphocytes (Figure 5(c)). A minor population of CD36+PD-1+ AT-SVF lymphocytes was further increased by western diet (Figure 5(d), p < 0.05), suggesting CD36 may regulate immune exhaustion during obesity and adipose inflammation.

## Discussion

Despite extensive investigations of adipose tissue immune cells, surprisingly little is known with respect to phenotypes and functions that distinguish adipose tissue-resident immune cells from those in other tissues such as blood and lymphoid organs. The present study suggests that CD36 may denote lymphocytes in adipose tissue and liver, organs with significant metabolic activity and functions that promote unique metabolic reprogramming of resident T cells.

The fatty acid transporter/scavenger receptor CD36 was upregulated in lymphocytes specifically in adipose tissue and liver of humans, monkeys, and mice, implying increased reliance on exogenous lipids for energy metabolism and homeostasis in these tissues. Although palmitate uptake (C16-BODIPY) was higher in CD36+ compared to CD36- AT-SVF lymphocytes in mice (Figure 5(b)), the functional role of adipose T cell CD36 requires further study as in vitro palmitate uptake by human AT-SVF lymphocytes showed high palmitate uptake despite treatment with a CD36 chemical inhibitor SSO (Supplementary Figure 1). However, the use of higher amounts of SSO and longer treatment times may be more effective [18]. Additionally, CD36 regulates uptake of different long-chain fatty acids such as oleate, and biochemical measurement of free fatty acids may better clarify the role of CD36-mediated fatty acid uptake by adipose lymphocytes. The high uptake of palmitate by adipose T cells in our study is consistent with the data of Han et al. who used the same assay to demonstrate significantly higher uptake of palmitate by adipose CD8 T cells compared to spleen and intestinal T cells [8]. The mechanisms that induce CD36 upregulation in T cells are also unclear. Despite a number of treatments that induce CD36 in other cell types, we did not observe CD36 induction in human blood T cells cultured with similar treatments (data not shown), highlighting the complexity of CD36 regulation in fat and liver T cells. Additionally, our observation of minimal to absent expression of CD36 by circulating T cells, especially when including a platelet marker such as CD41, is consistent with the study of Zamora et al. who showed that the presence of CD36 on blood T cells is likely due to platelet contamination [5].

Whether CD36 is preferentially expressed by a specific T cell subset (Th1, Th2, or Treg) in adipose tissue and liver is yet unclear. However, the CD36 upregulation was not specific to T cells in adipose tissue, as CD3-CD4- lymphocytes (likely NK cells and B cells), also upregulated CD36. Intriguingly, CD36 appeared to be expressed mostly by resting adipose T cells that did not express HLA.DR, CD38, or PD-1 (Figure 4) in monkeys, suggesting CD36 signalling may promote T cell functional quiescence in adipose tissue. Additionally, a population of CD36+PD-1+ lymphocytes were expanded in adipose tissue of mice during western diet (Figure 5(d)), indicating a role of CD36 for immune exhaustion, and which may have relevance to the increase of CD4+CD44+PD-1+ T cells in adipose tissue of high-fat diet mice observed by Shirakawa et al. [19]. The CD36+ adipose T cells may also include fat-resident Tregs, as Cipolletta et al. have shown that a high proportion of visceral adipose tissue CD3+CD4+Foxp3+ Tregs express CD36 in mouse models of obesity [6]. Additionally, Geys et al. showed that CD36-deficient mice have less Treg depletion in adipose tissue during high-fat diet compared to wild-type mice [7]. Lastly, whether adipose tissue T cells include Trm subsets like those of intestines and skin is unclear as we did not observe expression of CD103 in human adipose T cells (data not shown), consistent with the lack of CD103 expression by adipose T cells of rhesus monkeys reported by Han et al. [8]. CD69 is also considered a marker for Trm cells, but CD69 is widely expressed by T cells in most extravascular lymphoid and non-lymphoid tissues and not as unique to adipose T cells.

Limitations of the present study include the lack CD36 mRNA expression data, examination of T cell CD36 expression in other tissues such as intestines, skin, and skeletal muscle, lack of adipose macrophage examination for CD36 expression, and the role of soluble CD36. It is possible that circulating T cells may express some level of CD36 mRNA, which becomes upregulated following migration into adipose tissue, as Han et al. showed that CD36 mRNA in adipose T cells is significantly higher relative to spleen T cells in mice [8]. Additionally, Pan et al. showed significant CD36 mRNA upregulation in skin Trm cells following viral challenge in mice, further suggesting that Trm cells in other tissues such as intestines, lungs, and skeletal muscles may also express CD36 [9]. With respect to macrophages, CD36 would not be a useful marker for adipose tissue residence as CD36 is widely expressed by tissue macrophages and virtually all circulating monocytes express CD36. Lastly, CD36 is detectable in soluble form in serum and plasma, but may be mainly associated with exosomes, as Alkhatatbeh et al. showed that platelet-derived CD36+ microparticles accounted for the vast majority of plasma CD36 [20–23]. It is possible that adipocytes and adipose-resident macrophages secrete CD36+ microparticles that bind to adipose T cells and regulate functions.

CD36 expression and signalling are involved in inflammation and disease pathologies. We observed that CD36 expression in adipose T cells was increased in mice fed a western diet (Figure 5(a)), which may have relevance to the findings that associate higher expression of CD36 in adipocytes and adipose tissue macrophages to obesity and inflammation [10–14]. Recent studies have also shown that CD36 expression and fatty acid uptake in cancer cells can be increased by adipocytes and high-fat diet, resulting in significantly enhanced survival and metastases [15–17]. Determining the role of CD36 in the regulation of adipose tissue T cell metabolism and function, and whether this receptor represents a therapeutic target, merits further investigation.
