# Supplementary material for: Lymphocytes upregulate CD36 in adipose tissue and liver
Source: Adipocyte. 2019 Apr 30;8(1):154–63. doi: 10.1080/21623945.2019.1609202 (PMC6768236; doi:10.1080/21623945.2019.1609202)
Supplement: Supplemental Material [file kadi-08-01-1609202-s001.pptx]

## Slide 1
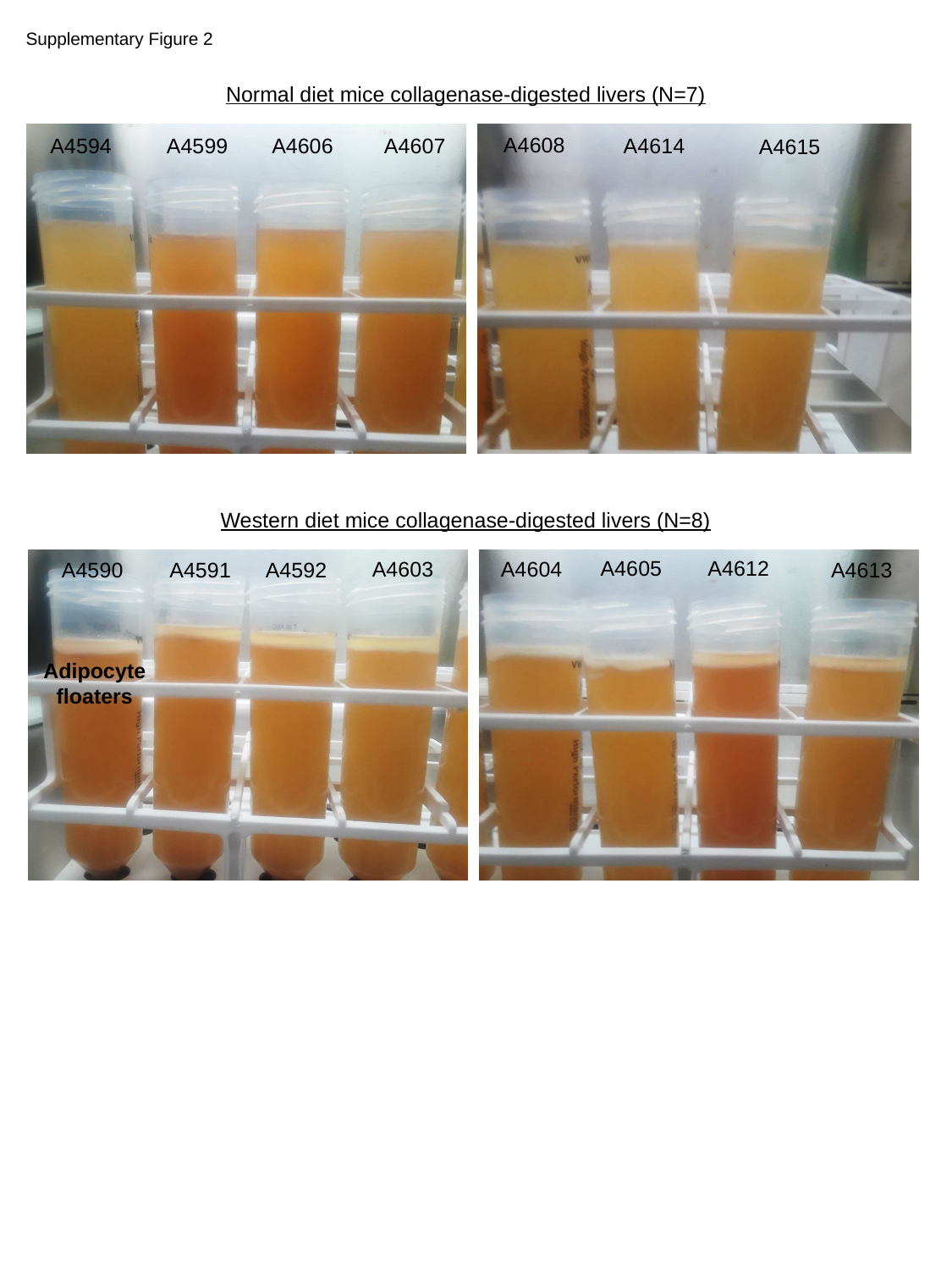

Supplementary Figure 2
Normal diet mice collagenase-digested livers (N=7)
A4608
A4607
A4614
A4594
A4599
A4606
A4615
Western diet mice collagenase-digested livers (N=8)
A4612
A4605
A4604
A4603
A4592
A4613
A4590
A4591
Adipocyte
floaters
