# Supplementary material for: Lymphocytes upregulate CD36 in adipose tissue and liver
Source: Adipocyte. 2019 Apr 30;8(1):154–63. doi: 10.1080/21623945.2019.1609202 (PMC6768236; doi:10.1080/21623945.2019.1609202)
Supplement: Supplemental Material [file kadi-08-01-1609202-s002.pptx]

## Slide 1
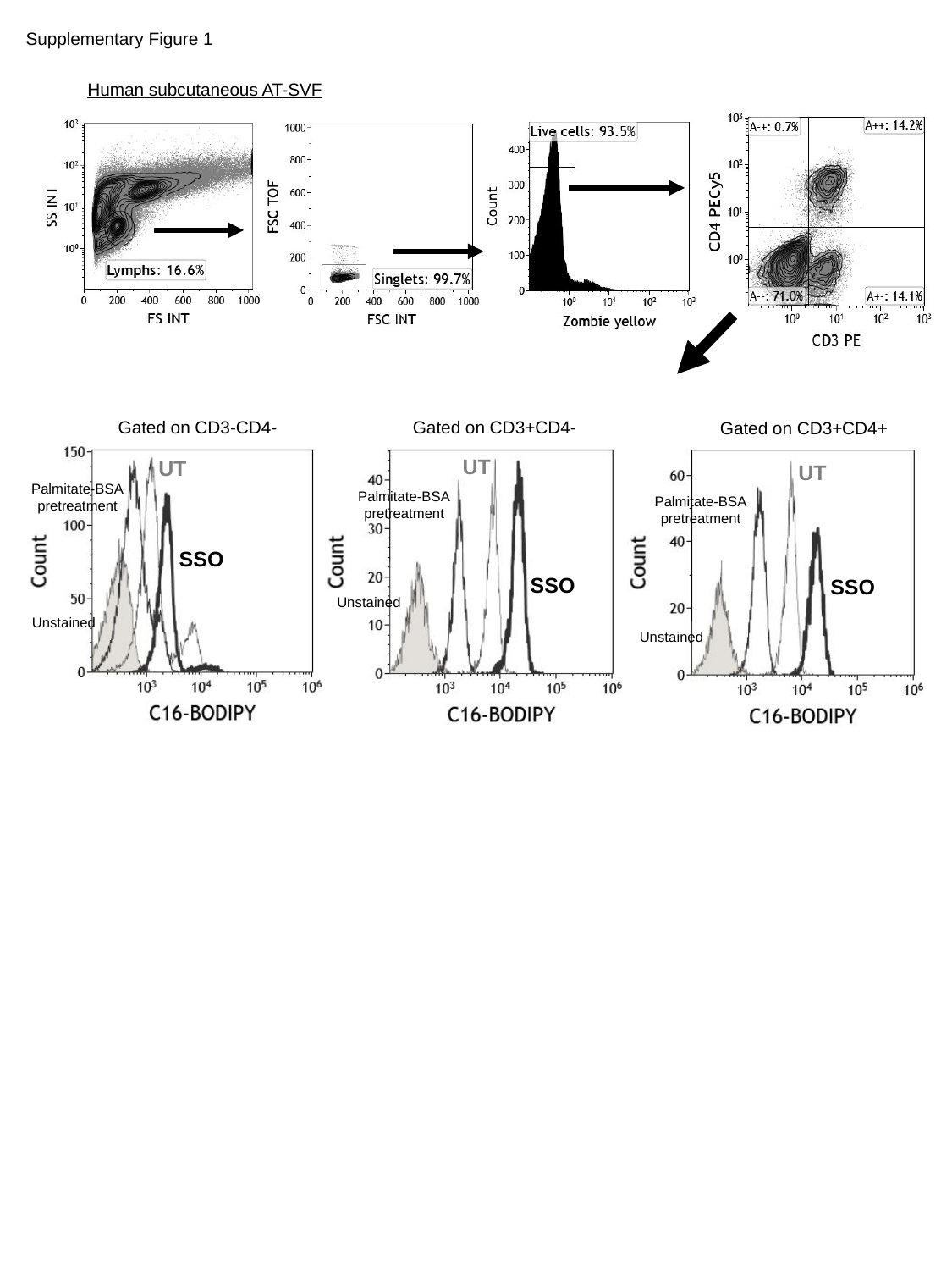

Supplementary Figure 1
Human subcutaneous AT-SVF
Gated on CD3-CD4-
Gated on CD3+CD4-
Gated on CD3+CD4+
UT
UT
UT
Palmitate-BSA
pretreatment
Palmitate-BSA
pretreatment
Palmitate-BSA
pretreatment
SSO
SSO
SSO
Unstained
Unstained
Unstained
